# Major cell types in the coronary thrombosis of acute myocardial infarction patients revealed by scRNA‐seq

**DOI:** 10.1002/ctm2.70181

**Published:** 2025-01-13

**Authors:** Zhiyao Wei, Cheng Cui, Zixuan Li, Jianping Li, Yibing Shao, Jizheng Wang, Jincheng Guo, Lei Song

**Affiliations:** ^1^ State Key Laboratory of Cardiovascular Disease Fuwai Hospital National Center for Cardiovascular Diseases Chinese Academy of Medical Sciences and Peking Union Medical College Beijing China; ^2^ Division of Cardiology Fuwai Hospital National Center for Cardiovascular Disease Chinese Academy of Medical Sciences and Peking Union Medical College Beijing China; ^3^ Division of Cardiology Beijing Luhe Hospital Capital Medical University Beijing China; ^4^ Division of Cardiology Peking University First Hospital Beijing China; ^5^ Division of Cardiology Qingdao Municipal Hospital Qingdao China

1

Dear Editor,

Intracoronary thrombosis, resulting from the rupture or erosion of atherosclerotic plaques, is the main cause of acute myocardial infarction (AMI). The role of immune cells in thrombosis has garnered attention as a central contributor to this catastrophic event.^1^ To further our understanding, we conducted single‐cell RNA sequencing to picture a cellular atlas of aspired intracoronary thrombus.

Our dataset comprised a total of 10 456 cells, and a median of 2786 genes were detected per cell (Figure 1). Unsupervised clustering revealed that the largest part of cells in the intracoronary thrombus were monocytes (29.3%). We further subclustered monocytes and identified eight subclusters (Figure 2A). Cluster 6 was identified as conventional dendritic cells, for the high expression levels of HLA‐DRB1 and HLA‐DRA. The remaining clusters were categorized based on the relative expression levels of CD14 and FCGR3A, leading to three classifications: classical (CD14^++^/FCGR3A^−^; clusters 0, 2, 4), intermediate (CD14^++^/FCGR3A^+^; clusters 1, 3) and non‐classical monocytes (CD14^+^/FCGR3A^++^; clusters 5, 7) (Figure 2B,C). Marker genes and functional heterogeneity for each cluster are presented in Figure 2D,E. Besides, among the monocytes, 14 modules of co‐regulated genes were identified by weighted gene co‐expression network analysis (WGCNA) (Figure 2F).

**FIGURE 1:**
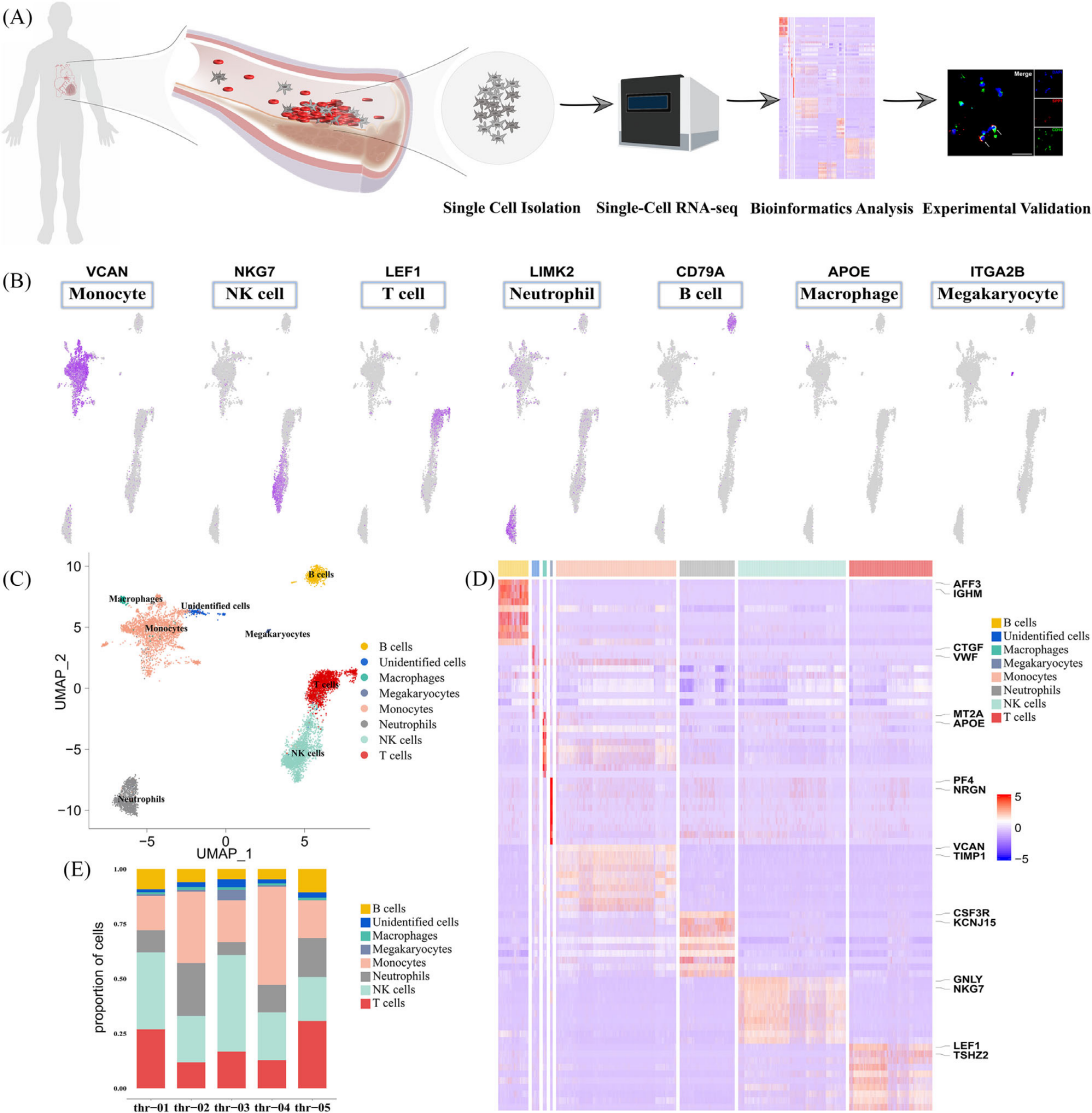
Single‐cell transcriptomic landscape of intracoronary thrombus. (A) Illustrator and workflow for single‐cell RNA sequencing. (B) Expression distribution of the marker gene(s) for each cell type. (C) UMAP visualization of cellular identity: monocytes (29.3%), NK cells (26.3%), T cells (20.3%), neutrophils (13.4%), B cells (7.4%), macrophages (1.0%) and megakaryocytes (.6%). A subset of cells lacking lineage‐specific markers accounted for 1.7% of all cells. (D) Heat map of top markers for each cell cluster. (E) Cell type profiles of each sample. UMAP, uniform manifold approximation and projection.

**FIGURE 2:**
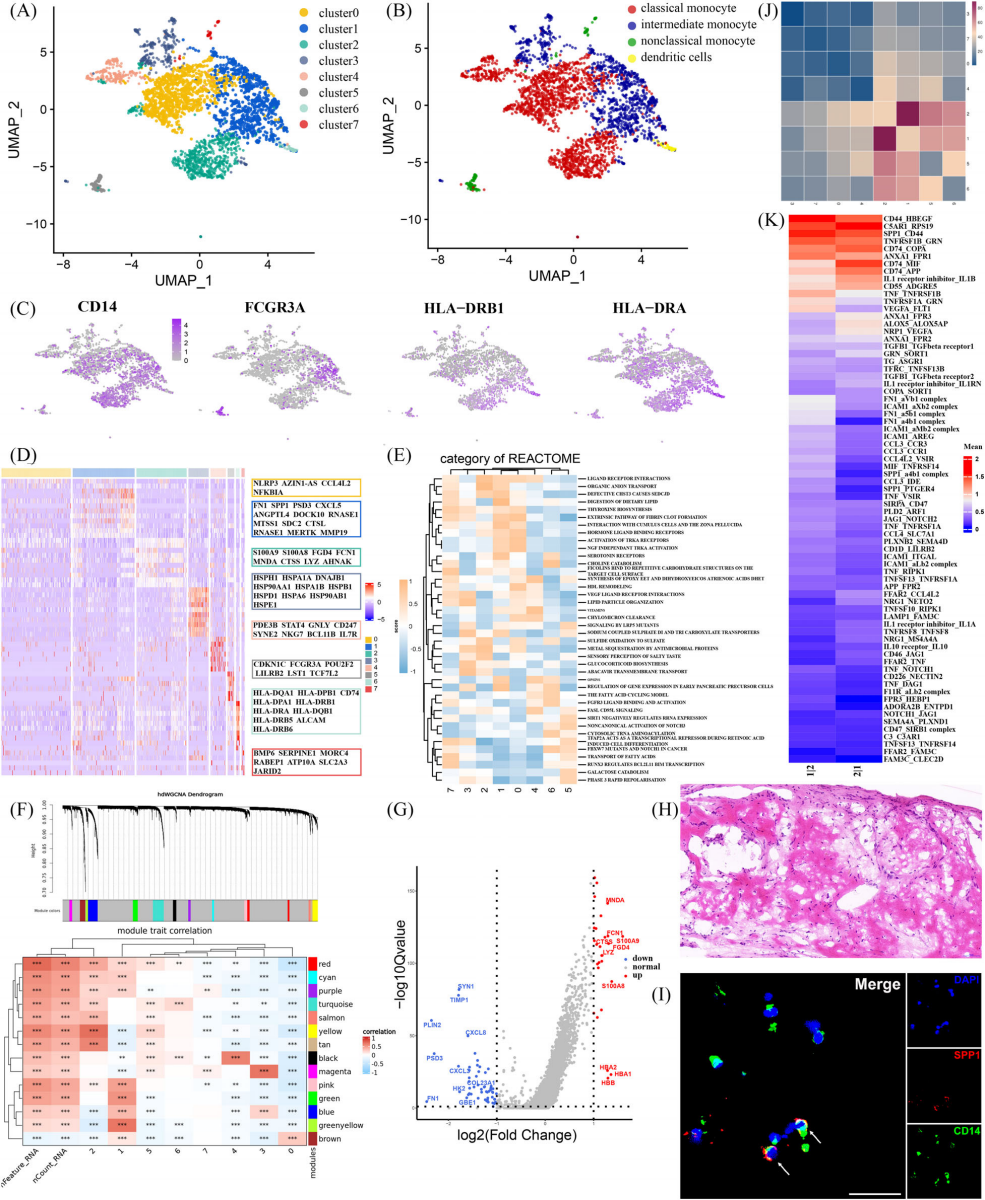
Monocyte clusters in intracoronary thrombus. (A) UMAP visualization of monocytes. (B) Classification of monocyte subclasses (classical, intermediate and non‐classical). (C) Feature plots for relative expression of CD14, CD16, HLA‐DRB and HLA‐DRA1. (D) Heat map of top markers for each cell cluster. (E) Differential pathway activities analysed by GSVA. (F) WGCNA‐derived gene co‐expression modules with correlation heat map. (G) Volcano plot of differentially expressed genes in cluster 2. (H) Haematoxylin and eosin staining of segmental material from intracoronary thrombus. (I) Immunofluorescence staining for CD14 (green), and SPP1 (red) performed on a serial section from the same sample. DAPI was used for nuclear counterstain. Histological analyses of the intracoronary thrombus revealed a prominent representation of cellular components, with staining for SPP1 and CD14 confirming the presence of SPP1^+^ intermediate monocytes. GSVA, gene set variation analysis; UMAP, uniform manifold approximation and projection; WGCNA, weighted gene co‐expression network analysis.

Classical monocytes comprised the largest proportion of the monocyte population (60.1%) and exhibited diverse functions. Cluster 0 (30.1%) demonstrated significant activation in response to non‐infectious stimuli during AMI, characterized by elevated expression levels of NLRP3, IL‐1B and NFKBIA. Module brown (TNF and NF‐kappa signalling pathway) displayed a strong correlation with cluster 0 (Figure S1A). Cluster 2 (21.8%) displayed heightened expression of inflammatory mediators including S100A9, S100A8, CTSS and phagocytosis receptor CD36 (Figure 2G). Figure 2H,I proved its presentation. This cluster had a strong correlation with module tan, which is linked to phagosome and leukocyte trans‐endothelial migration (Figure S2B). Cluster 4 (6.6%) exhibited elevated expression of T cell activation genes (CD247, NKG7, GNLY) and was associated with module black (nature killer [NK]‐mediated cytotoxicity) (Figure S1C).

Intermediate monocytes represented 35.7% of the total monocyte population and comprised two distinct clusters. Cluster 1 (26.9%) showed a pro‐fibrotic and anti‐inflammatory profile, characterized by high expression levels of genes associated with fibrosis (FN1, SPP1), cardiac protection (CTSL, ANGPTL4), T cell activation (ALCAM), oxidized low‐density lipoprotein‐induced cell injury (ANGPTL4) and phagocytosis (MERTK). Four modules were predominantly expressed in cluster 1. Notably, module pink facilitated substrate adhesion‐dependent cell spreading and endocytosis (Figure S1D), while module green‐yellow was associated with focal adhesion, extracellular matrix−receptor interaction and calmodulins (Figure S1E). Intercellular communication network analysis among monocytes indicated a robust connection between clusters 1 and 2 (Figure 2J). Close receptor‐ligand pairs included SPP1‐CD44 and MIF‐CD74 (Figure 2H), both associated with immunosuppression and the M1/M2 macrophage transition.^2^, ^3^ Cluster 3 (8.8%) exhibited an activated cellular response to heat stress, as evidenced by the high expression of heat shock proteins and the activation of MAPK signalling pathway, indicating its role as a stress‐reacted protective monocyte subtype. Module magenta (regulation of cellular response to heat, response to unfolded protein, DNA repairment and IF‐8 production) showed preferential expression in cluster 3 (Figure S1F). Additionally, module blue (HIF‐1 signalling pathway and glycolysis/gluconeogenesis) reflected metabolic reprogramming in inflammatory macrophages responding to tissue injury in a hypoxic environment^4^ (Figure S1G), significantly correlating with both intermediate monocyte clusters.

Non‐classical monocytes, comprising clusters 5 and 7, constituted 4.2% of the total monocyte population. Cluster 5 (2.8%) expressed genes associated with patrolling (VAV2, ITGAL, PKN1 and ARHGEF18, Figure 3A), and showed a predominant role in haemostasis. Upregulated functions included platelet activation, signalling and aggregation, regulation of cell shape, cell surface interactions at the vascular wall and glycoprotein VI−mediated activation cascade (Figure 3B). Furthermore, it facilitated T cell proliferation, neutrophil degranulation, Fc epsilon RI‐mediated MAPK activation and cytokine signalling, highlighting its potential involvement in both innate and adaptive inflammatory reactions to plaque eruption/erosion during AMI. CDKN1C, a potent cell‐cycle inhibitor, was highly expressed in this subset (Figure 3C), indicative of its proapoptotic and highly differentiated nature. Cluster 7 (1.4%), the smallest monocyte cluster, exhibited pronounced expression of homeostasis‐related terms including VEGF signalling, and complement and coagulation cascades. Recruited monocytes are known to amplify thrombosis.^5^ Our study proved that non‐classical monocytes, previously thought to be associated with post‐AMI healing,^6^ in fact contributed to homeostasis and thrombosis.

**FIGURE 3:**
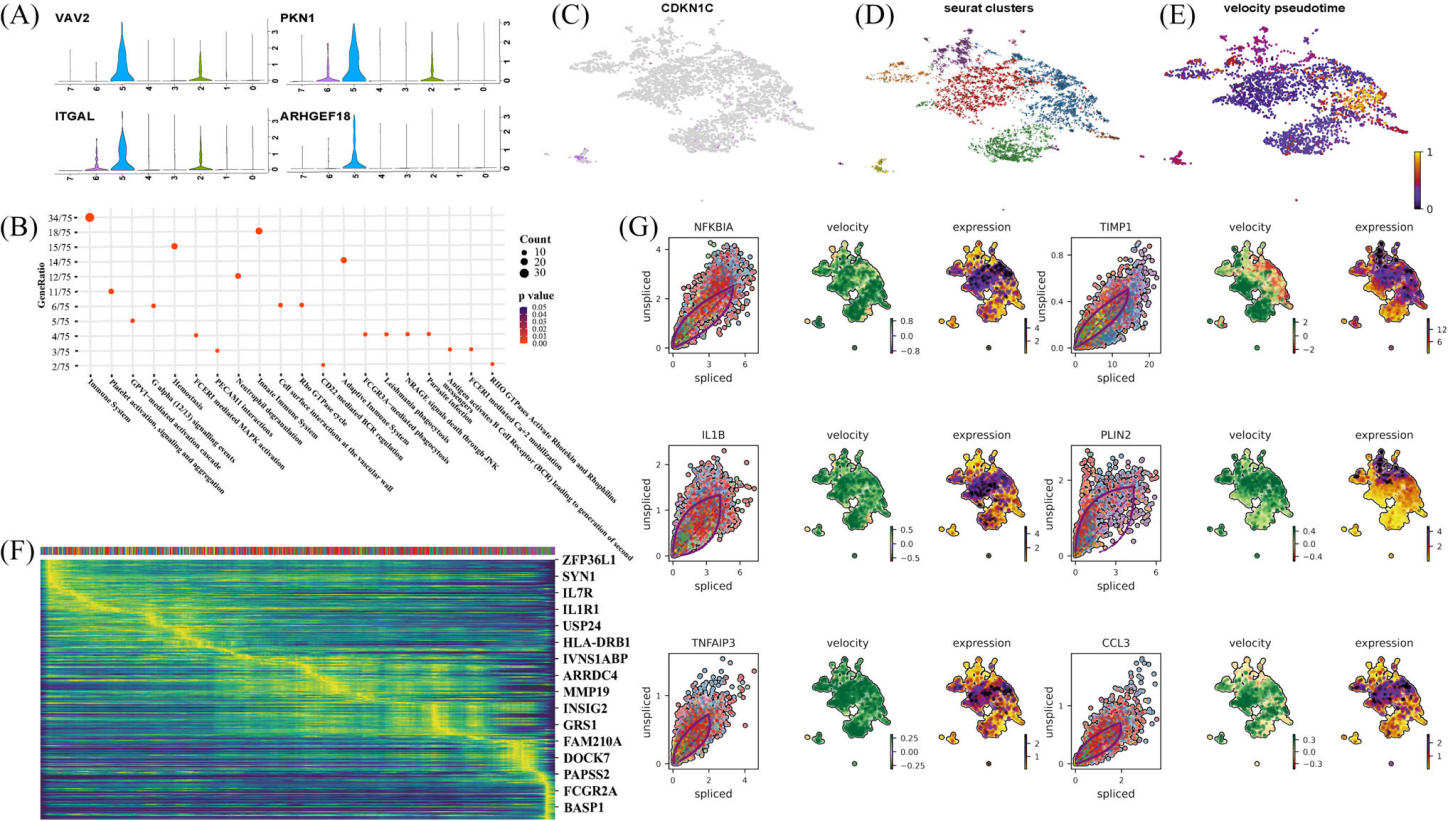
Transcriptomic profile of non‐classical monocyte clusters in intracoronary thrombus. (A) Violin plot of VAV2, ITGAL, PKN1 and ARHGEF18 in each cluster. (B) Reactome analysis of upregulated expression in cluster 5. (C) Feature plots for relative expression of CDKN1C. (D) Differentiation trajectory of monocytes. (E) Velocity pseudotime of monocytes. (F) Heatmap for expression dynamics during monocytes differentiation. (G) Spliced/unspliced percentage, velocity and expression of genes whose expression was dynamically changed over latent time.

To infer cellular state progression, RNA velocities were estimated (Figure 3D). Cluster 0 marked the starting point of pseudotime, representing the most immature monocyte cluster (Figure 3E), while cluster 1 denoted the most advanced stage of differentiation. Intermediate monocytes and other classical clusters differentiated from cluster 0, whereas non‐classical monocytes exhibited a more distinct trajectory. These findings suggest that intermediate monocytes arise locally from resident classical monocytes, whereas non‐classical monocytes may originate from circulating monocytes. The visualized velocity and the top six pseudotime‐dependent genes − including NFKBIA, TIMP1, IL1B, PLIN2, TNFAIP3 and CCL3 − were shown in Figure 3F,G, all of which underscore their pivotal role in the AMI process.^7^, ^8^, ^9^ Both NFKBIA and TNFAIP3 serve as negative inflammatory modulators. Moreover, TIMP1, PLIN2 and CCL3 have all been recognized as predictors of future cardiovascular events in AMI patients.

In addition to monocytes, neutrophils in the coronary thrombus were classified into three types (Figure S2). Cluster 1 (44.9%) represented N1 neutrophils in a heightened inflammatory state. Cluster 2 (2.6%) was a small subpopulation of exhausted neutrophils expressing GRK5.^10^ NK cells were subdivided into five clusters (Figure S3), with clusters 0, 1, 2 representing type II NKT cells, while clusters 3 and 4 comprised CD56^dim^ NK cells. T cells were categorized into five subclusters, with one identified as CD8^+^ T cells (cluster 2) and four as CD4^+^ T cells (clusters 0, 1, 3, 4) (Figure S4). Macrophages constituted a minor population in coronary thrombus and could be subclustered into two types (Figure S5): cluster 0 represented an anti‐inflammatory type, whereas cluster 1 exhibited an accelerating phenotype.

In conclusion, we found that SPP1^+^ intermediate monocytes constitute a large proportion of intra‐thrombus monocytes and play a significant role in fibrosis, anti‐thrombosis and metabolic reprogramming. Contrary to expectations, intra‐thrombus non‐classical monocytes act as pro‐thrombotic patrols, promoting atherothrombosis rather than exhibiting anti‐inflammatory effects. Key cell subclusters identified in this study provide potential therapeutic targets for cardiovascular ischemic events.

## AUTHOR CONTRIBUTIONS

Z.W. designed the project, analysed the data, interpreted the results and wrote the manuscript. C.C., Z.L., J.L. and Y.S. collected patient samples. J.W. performed nucleus isolation and snRNA‐seq. J.G. and L.S. supervised the project.

## CONFLICT OF INTEREST STATEMENT

The authors declare no conflict of interest.

## FUNDING INFORMATION

This work was supported by the Beijing Municipal Science and Technology Commission [grant number: Z191100006619106] and the National High Level Hospital Clinical Research Funding [grant number: 2023‐GSP‐QN‐3].

## ETHICS STATEMENT

The institutional review board central committee at Fuwai Hospital approved the study (2022‐1734), and all eligible patients provided informed consent.

## Supporting information



Supporting information

Supporting information

Supporting information

Supporting information

Supporting information

Supporting Information

## Data Availability

The datasets used and/or analysed during the current study are available from the corresponding author on reasonable request.

## References

[1] Stark K , Massberg S . Interplay between inflammation and thrombosis in cardiovascular pathology. Nat Rev Cardiol. 2021;18(9):666‐682. 33958774 10.1038/s41569-021-00552-1 PMC8100938

[2] Xing J , Cai H , Lin Z , et al. Examining the function of macrophage oxidative stress response and immune system in glioblastoma multiforme through analysis of single‐cell transcriptomics. Front Immunol. 2023;14:1288137. 38274828 10.3389/fimmu.2023.1288137 PMC10808540

[3] Ghoochani A , Schwarz MA , Yakubov E , et al. MIF‐CD74 signaling impedes microglial M1 polarization and facilitates brain tumorigenesis. Oncogene. 2016;35(48):6246‐6261. 27157615 10.1038/onc.2016.160

[4] Zhang S , Bories G , Lantz C , et al. Immunometabolism of phagocytes and relationships to cardiac repair. Front Cardiovasc Med. 2019;6:42. 31032261 10.3389/fcvm.2019.00042 PMC6470271

[5] Matter MA , Paneni F , Libby P , et al. Inflammation in acute myocardial infarction: the good, the bad and the ugly. Eur Heart J. 2024;45(2):89‐103. 37587550 10.1093/eurheartj/ehad486 PMC10771378

[6] Nahrendorf M , Swirski FK , Aikawa E , et al. The healing myocardium sequentially mobilizes two monocyte subsets with divergent and complementary functions. J Exp Med. 2007;204(12):3037‐3047. 18025128 10.1084/jem.20070885 PMC2118517

[7] Rizzo G , Gropper J , Piollet M , et al. Dynamics of monocyte‐derived macrophage diversity in experimental myocardial infarction. Cardiovasc Res. 2023;119(3):772‐785. 35950218 10.1093/cvr/cvac113 PMC10153424

[8] Nordeng J , Schandiz H , Solheim S , et al. TIMP‐1 expression in coronary thrombi associate with myocardial injury in ST‐elevation myocardial infarction patients. Coron Artery Dis. 2022;33(6):446‐455. 35102064 10.1097/MCA.0000000000001128

[9] Russo M , Montone RA , D 'Amario D , et al. Role of perilipin 2 in microvascular obstruction in patients with ST‐elevation myocardial infarction. Eur Heart J Acute Cardiovasc Care. 2021;10(6):633‐642. 33620432 10.1093/ehjacc/zuaa004

[10] Arraes SM , Freitas MS , da Silva SV , et al. Impaired neutrophil chemotaxis in sepsis associates with GRK expression and inhibition of actin assembly and tyrosine phosphorylation. Blood. 2006;108(9):2906‐2913. 16849637 10.1182/blood-2006-05-024638

