# Supplementary figures and images for: Major cell types in the coronary thrombosis of acute myocardial infarction patients revealed by scRNA‐seq
Source: Clin Transl Med. 2025 Jan 13;15(1):e70181. doi: 10.1002/ctm2.70181 (PMC11727574; doi:10.1002/ctm2.70181)

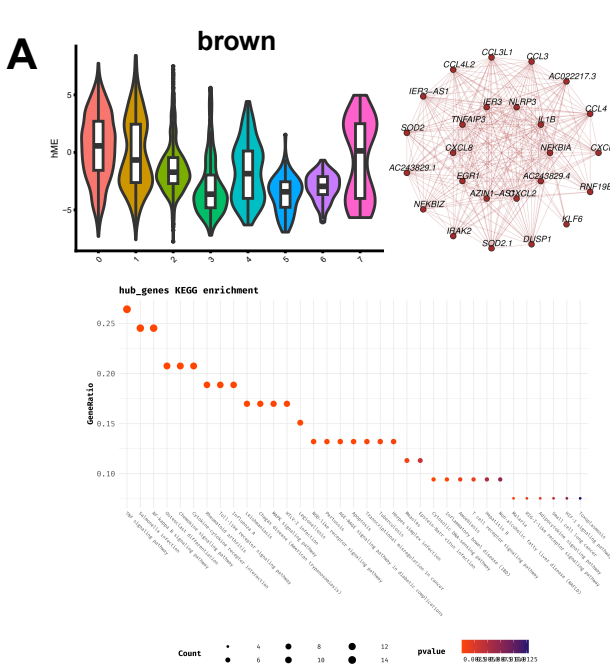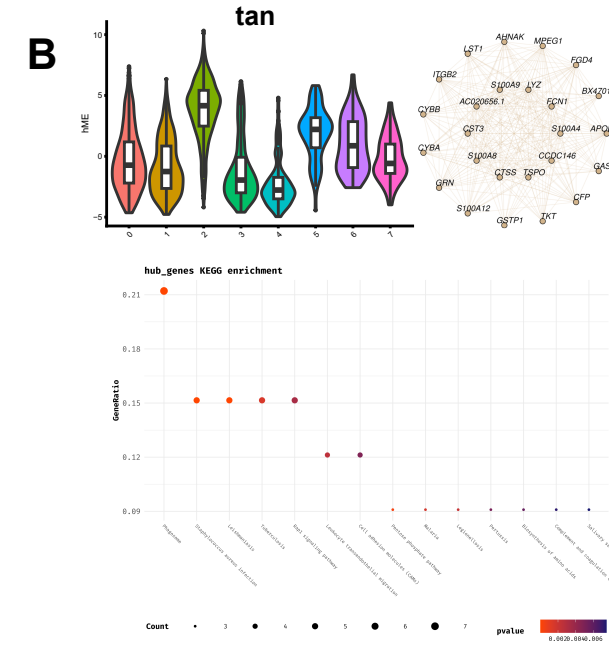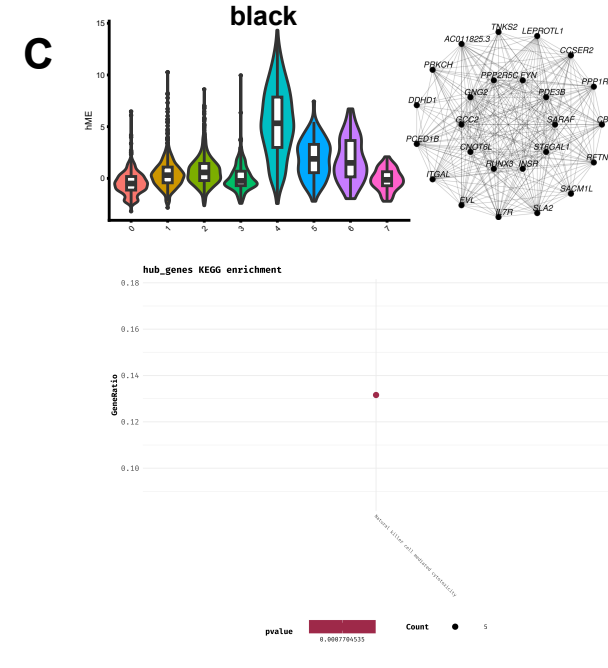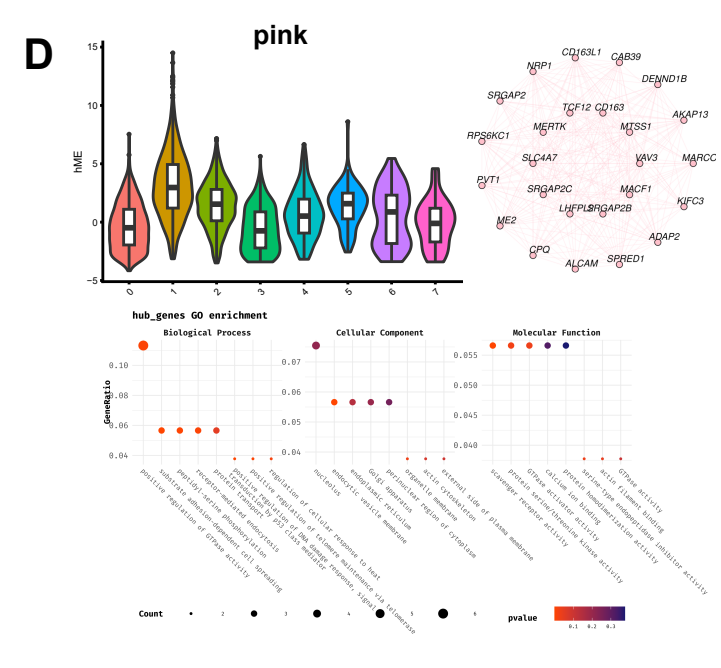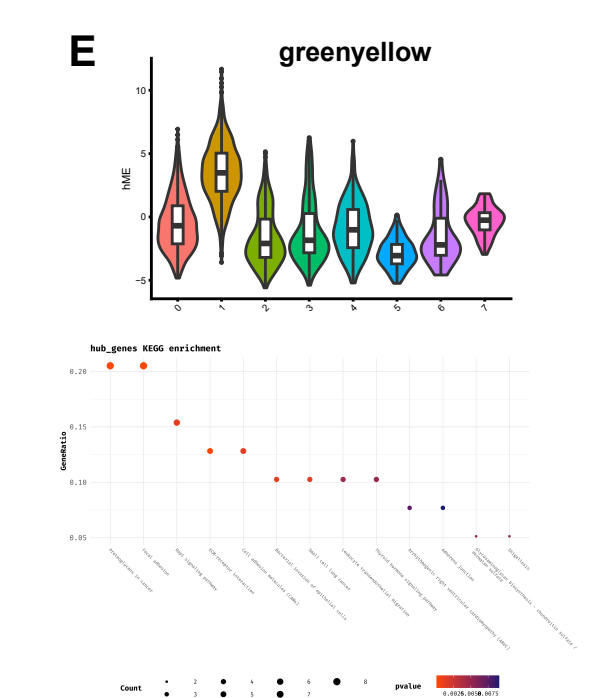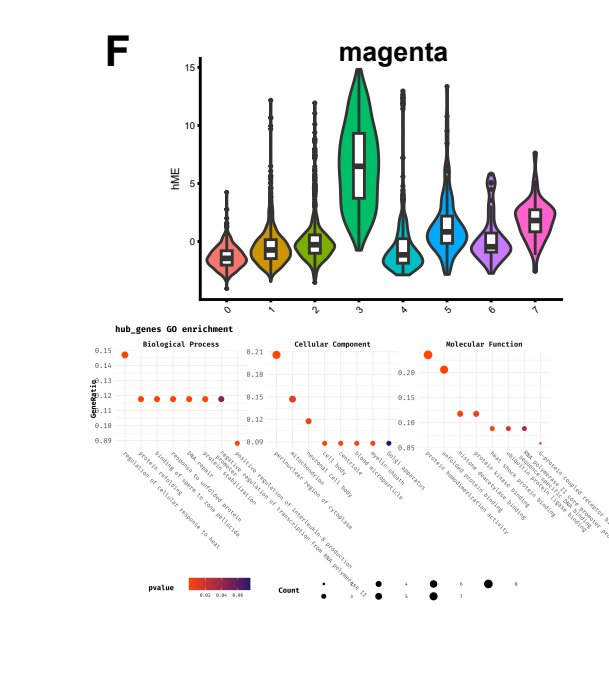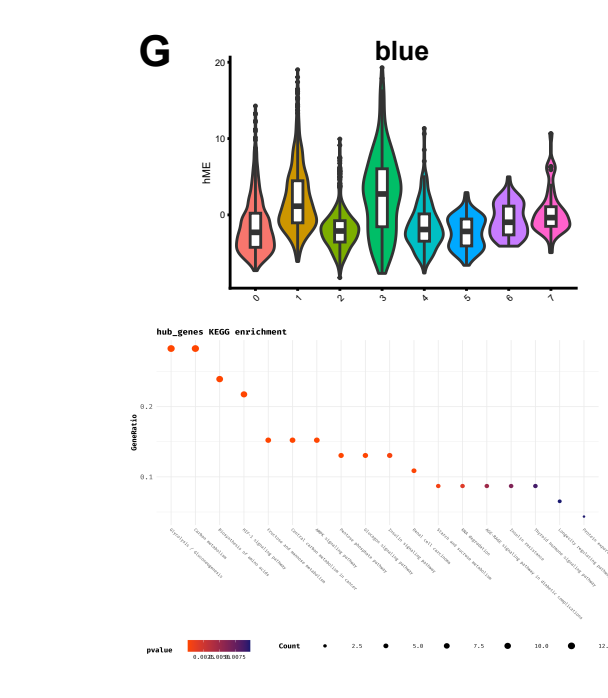

Supplement: Supplementary file 1 — Supporting information [file CTM2-15-e70181-s006.pdf]

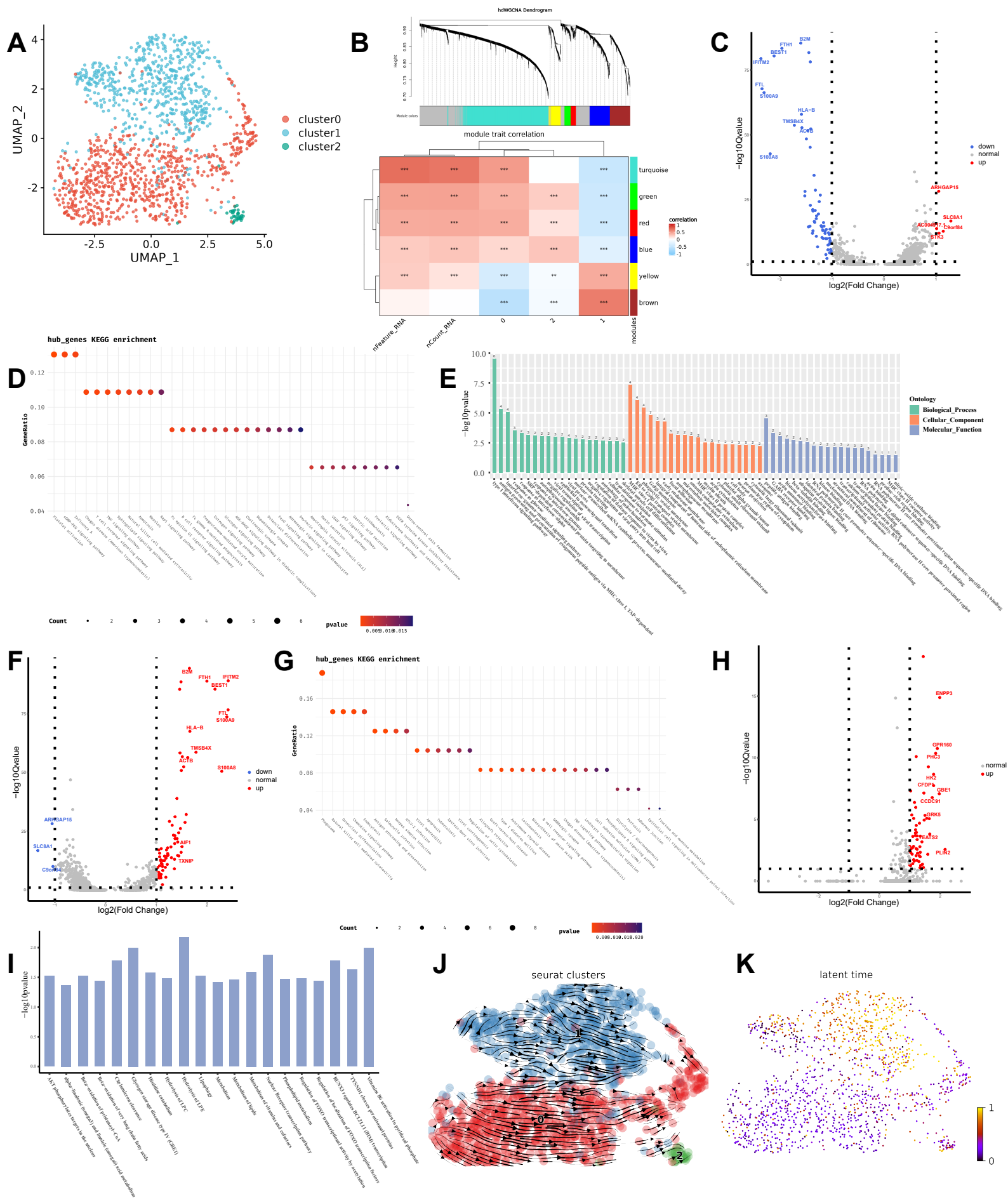

Supplement: Supplementary file 2 — Supporting information [file CTM2-15-e70181-s001.pdf]

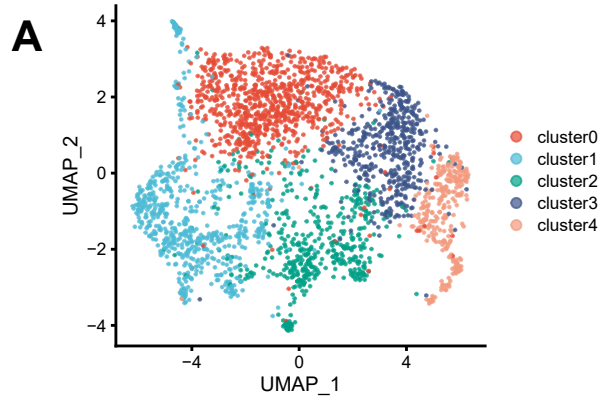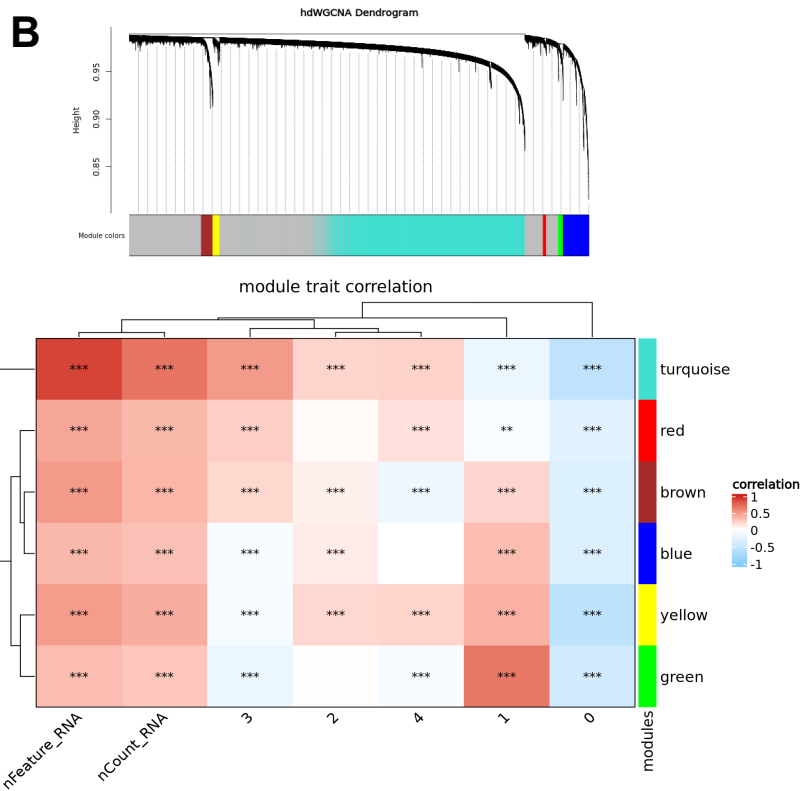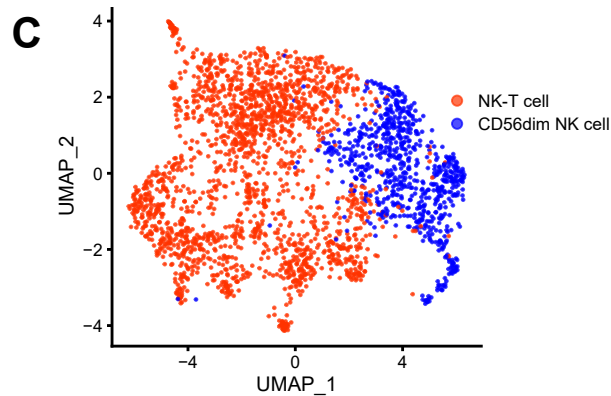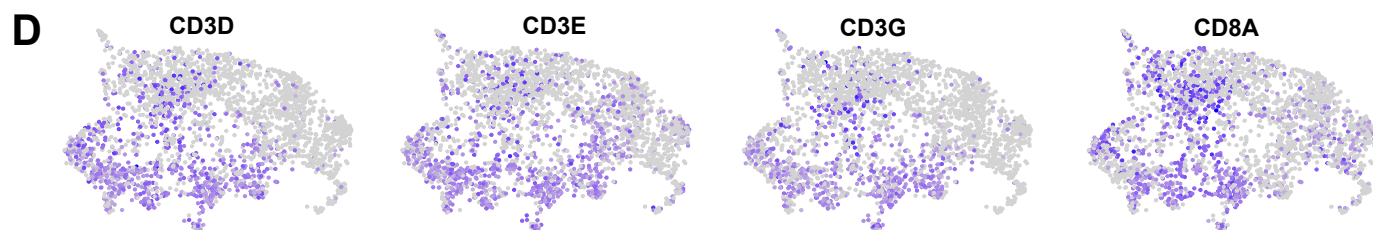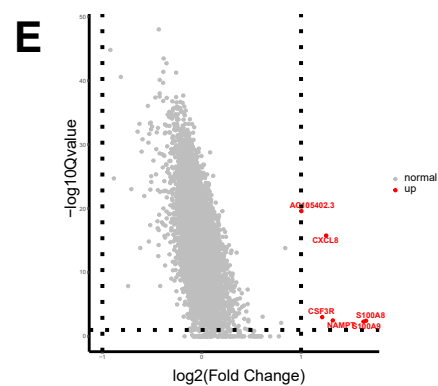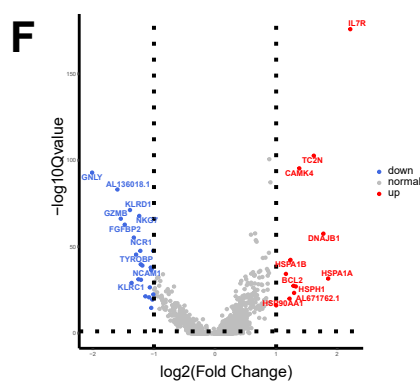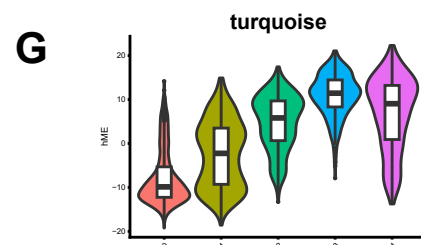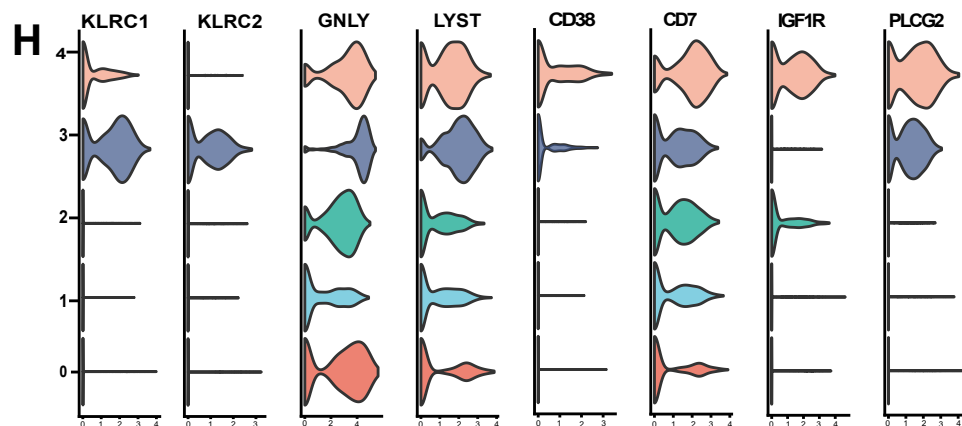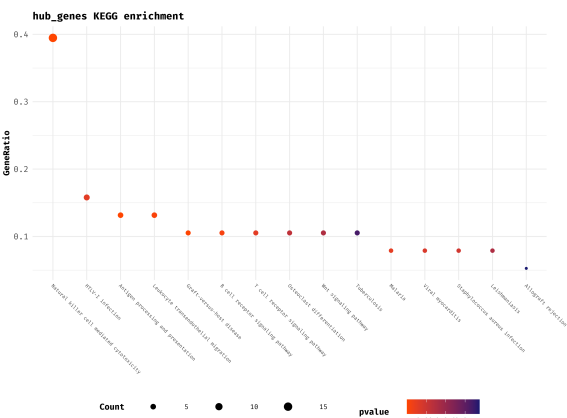

Supplement: Supplementary file 3 — Supporting information [file CTM2-15-e70181-s004.pdf]

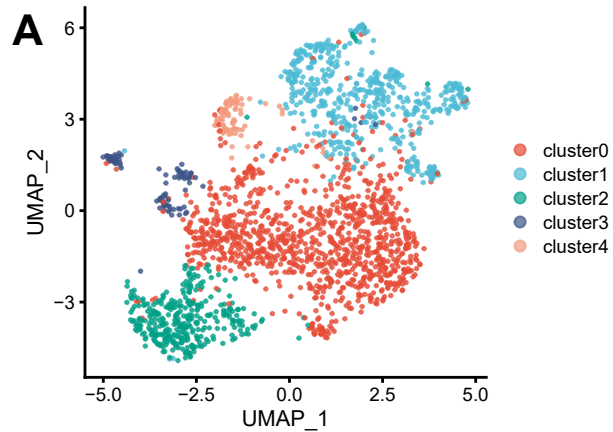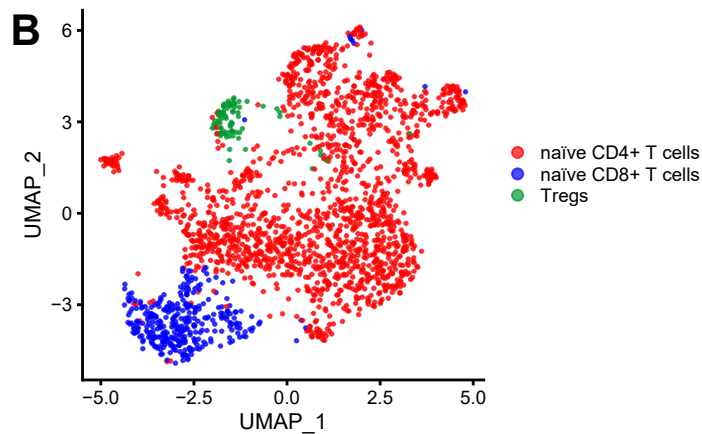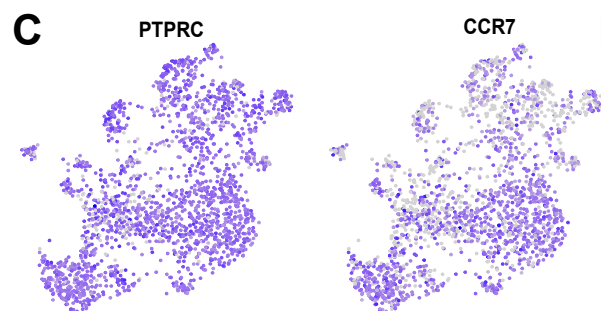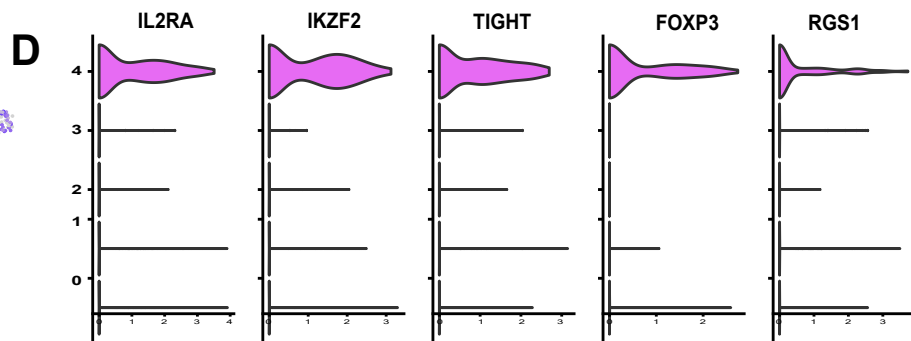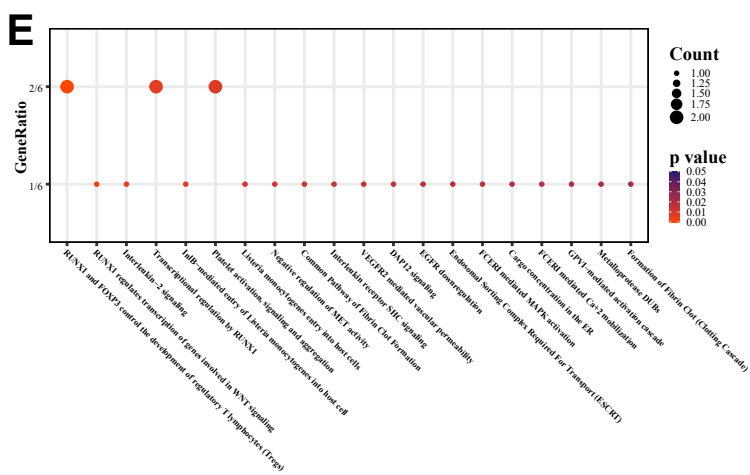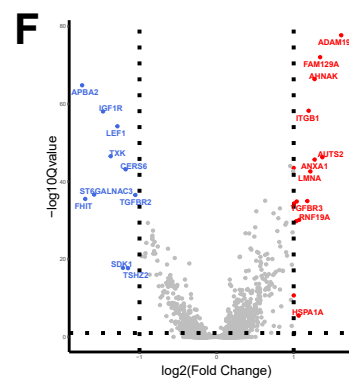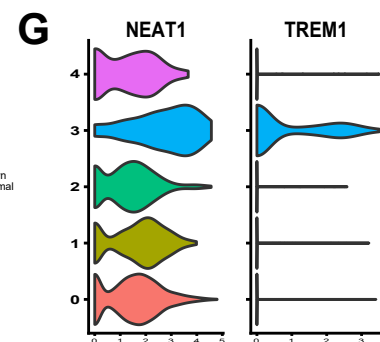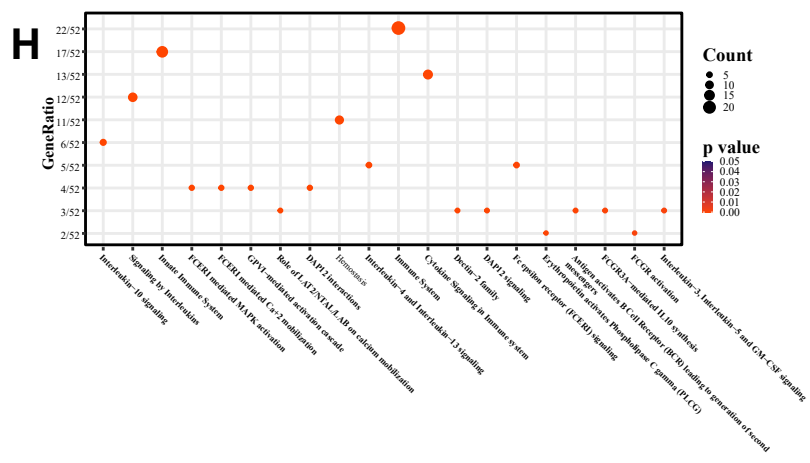

Supplement: Supplementary file 4 — Supporting information [file CTM2-15-e70181-s005.pdf]

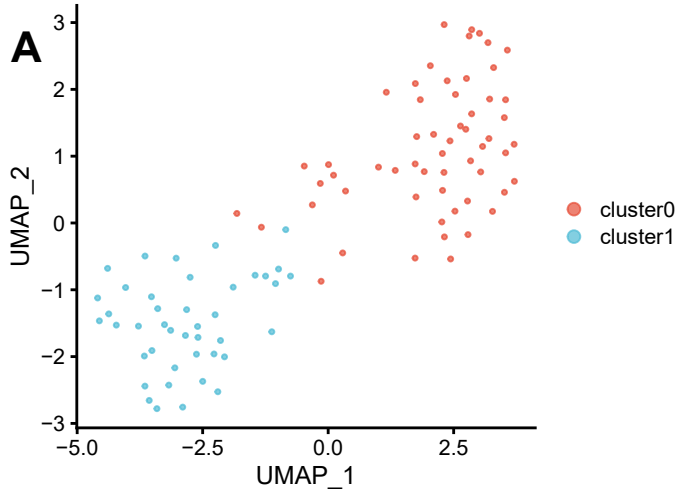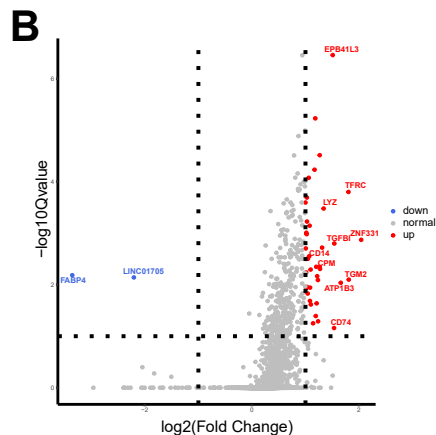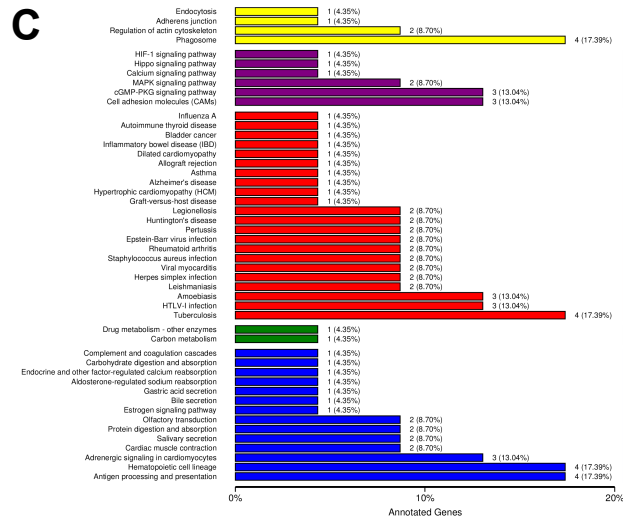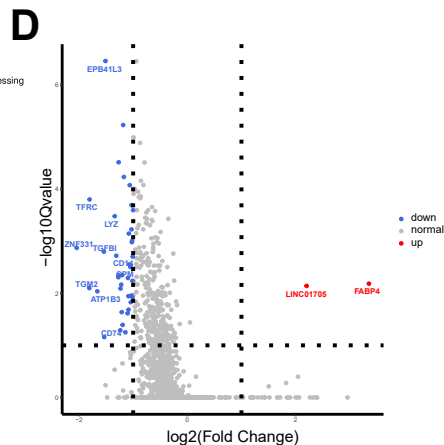

Supplement: Supplementary file 5 — Supporting information [file CTM2-15-e70181-s002.pdf]
