# Supplementary material for: Major cell types in the coronary thrombosis of acute myocardial infarction patients revealed by scRNA‐seq
Source: Clin Transl Med. 2025 Jan 13;15(1):e70181. doi: 10.1002/ctm2.70181 (PMC11727574; doi:10.1002/ctm2.70181)
Supplement: Supplementary file 6 — Supporting Information [file CTM2-15-e70181-s003.docx]

**Cell Dissociation from Atherothrombosis for scRNAseq.**

Specimens were thoroughly washed with 4 ℃ DPBS and digested at 37℃ for 1 hour using trypsin (2.5g/L). The resulting mixture was then filtered through 40µm cell strainers, washed in PBS, and centrifuged at 300g for 5 minutes. Red blood cells were removed by Red Blood Cell Lysis Buffer (Solarbio, R1010), dead cells were removed by Dead Cell Removal Kit (MACS, 130-090-101), and cell debris was removed by Debris Removal Solution (MACS, 130-109-398) following the manufacturer’s protocols.

**scRNA-Seq Library Preparation and Sequencing**

We utilized the BMKMANU DG1000 Library Construction Kit (BMKMANU) based on droplet microfluidics technology for library construction^12,13^. Specifically, each library contained an average of 4,271 cells with a concentration of 1,050.6 cells/μL and an activity exceeding 92%. Cells were encapsulated into droplets, where cell lysis and mRNA capture were performed using the DG1000 portable single cell system. Single-cell microdroplets were recovered via the emulsion breaking recovery system, followed by reverse transcription of magnetic bead-captured mRNA into cDNA and cDNA amplification. The final libraries were sequenced on the Illumina NovaSeq6000 system, generating 200G paired bases in PE150 mode in total.

**Raw Data Processing**

Sequencing results were demultiplexed and converted to FASTQ format using Illumina bcl2fastq software. Sample demultiplexing, barcode processing and single-cell gene counting were performed using BSCMatrix (v2.1). scRNA-seq data were aligned to the reference genome, and cells meeting the criteria of 500 to 7,000 expressing genes, ≥100 unique molecular identifiers (UMI), and <20% mitochondrial counts were retained. Cells exhibiting high expression of HBA, HBB, and HBD, suggestive of red blood cells, were excluded from further analysis. Doublets were identified and removed using the DoubletFinder package (v2.0.3).The Uniform Manifold Approximation and Projection (UMAP) algorithm was employed to project cells into two-dimensional space, with clustering parameter resolution set to 0.2 for identifying cell clusters annotated by marker genes. Analysis was performed using the R package Seurat (v 4.0.3).

**RNA Velocity Analysis**

RNA velocity analysis was conducted using Velocyto (v0.17.17) and scVelo (v0.2.4). This method estimated RNA velocities of individual cells by distinguishing between unspliced and spliced mRNAs, thereby visualizing cellular dynamics through streamlines. Genes with high probabilities in the dynamic model were considered potential driver genes , while latent time was used to reconstruct the temporal sequence of transcriptomic events.

**Intercellular Communication**

Interactions between ligands and receptors across cell types were identified utilizing CellPhoneDB (v4). The potential strength of interactions between cell types was determined using probability values of communication. Interaction scores between subclusters was determined by a specific ligand-receptor pair based on average gene expression of the ligand in one subcluster and the receptor in another. To identify significant cell-cell interactions, we permuted cell type labels 1000 times to calculate the significance of each pair.

**Weighted Gene Co-expression Network Analysis**

We utilized the R package hdWGCNA (v0.3.00) for weighted co-expression network analysis (WGCNA) to identify modules of highly co-expressed genes.

**Gene Set Variation Analysis**

Gene Set Variation Analysis (GSVA) was performed using GSVA (v1.52.3) to assess the pathway activity within distinct cell clusters. This nonparametric, unsupervised method converted gene expression matrices into gene sets, providing pathway activity scores for evaluating pathway activities within clusters.

**Immunofluorescence staining**

1. μm-thick frozen sections obtained from intracoronary thrombus were immersed in cold ethanol for 10 min, blocked with 3% normal serum for 2 hours at room temperature. The sections were then incubated overnight in a humidified chamber at 4 °C with primary antibodies: rabbit monoclonal anti-SPP1 (1:50; ab214050, Abcam) and mouse monoclonal anti-CD14 (1:50; ab181470, Abcam). Goat anti-rabbit 488- and anti-mouse 594 conjugated secondary antibodies (1:200; Beyotime) were added and incubated for 1 hour at room temperature.

The basic epidemiological characteristics of the patients (age, sex), as well as previous inflammatory diseases and time of infarct evolution should be reported, as these may influence the results

**Table S1**. Clinical characteristics of involved patients

| Sample ID | Age (y) | Sex | Comorbidities | Onset to guidewire passage (h) | Time of onsert | Time of guidewire passage |
| --- | --- | --- | --- | --- | --- | --- |
| thr-01 | 41 | Male | Hypertension | 3.0 | 2022-10-15 0:00 | 2022-10-15 3:00 |
| thr-02 | 48 | Male | None | 2.6 | 2023-2-22 17:00 | 2023-2-22 19:36 |
| thr-03 | 56 | Male | None | 2.8 | 2023-02-16 9:40 | 2023-02-16 12:27 |
| thr-04 | 42 | Male | None | 17.8 | 2023-02-14 18:30 | 2023-02-15 12:17 |
| thr-05 | 37 | Male | Hypertension | 12.7 | 2023-03-02 04:00 | 2023-03-2 16:40 |

**Table S2.** Sequencing information of each sample

| Sample | Median genes per cell | Median UMI counts per cell | Total gene detected | Total cell number |
| --- | --- | --- | --- | --- |
| thr-01 | 2050 | 4987.5 | 23624 | 3620 |
| thr-02 | 2864 | 8658 | 19165 | 899 |
| thr-03 | 4771 | 37250.5 | 12121 | 84 |
| thr-04 | 687 | 1319 | 18399 | 4263 |
| thr-05 | 2783.5 | 8350 | 20907 | 1590 |
